# Is unilateral transverse process-pedicle percutaneous kyphoplasty a better choice for osteoporotic thoracolumbar fractures in the old patients?

**DOI:** 10.1186/s12893-021-01246-8

**Published:** 2021-05-21

**Authors:** Wu Tao, Qin Hu, Yap San Min Nicolas, Xu Nuo, Huang Daoyu, Jin Zhen, Sun Jinpeng, Liu Jun

**Affiliations:** Orthopedics Department, The Second Affiliated Hospital of Nanjing Medical University, Jiangjiayuan Road No. 121, Nanjing, 210000 Jiangsu Province China

**Keywords:** Osteoporotic vertebral compression fractures, Osteoporosis, Percutaneous kyphoplasty, Surgical approach, Pain relief, Cement leakage

## Abstract

**Background:**

A few reports have shown that unilateral transverse process-pedicle percutaneous kyphoplasty is a good choice for patients with osteoporotic vertebral compression fracture (OVCF). However, this issue remains controversial and the related comprehensive research was lacked.

**Methods:**

A retrospective study was conducted on patients receiving PKP surgery for OVCF. Patients were divided into three groups according to surgical approach. Symptom and radiographical evaluation were performed preoperatively, 1-month postoperatively, 1-year postoperatively and follow-ups. And follow-ups were repeated every year. Visual Analogue Scale Score (VAS), Oswestry Disability Index (ODI) scores, anterior vertebral height, coronal Cobb angle and sagittal Cobb angle was determined and compared among three groups.

**Results:**

Totally 447 patients were included with an average age of 76.67.2years old. UTP showed significantly shorter surgical duration (p<0.001), lower cement volume (p<0.001) but higher cement leakage proportion (p=0.044). No significant statistical difference was found in terms of improvement rates among three groups. Besides, it was notable that the a significantly higher coronal Cobb angle was observed in UTP group, and a about 4coronal correction was found after UTP PKP.

**Conclusion:**

UTTP PKP could achieve similar symptoms relief and kyphosis correction as UTP and BTP PKP. However, it had shorter surgical time and less radio exposure than BTP PKP, lower risk of cement leakage and higher proportion of bilaterally cement distribution than UTP PKP. It seemed to be a better choice for patients with OVCF. In addition, we found that UTP PKP was especially fit for OVCF patients with asymmetrical vertebral compression.

## Background

With the social development and medical innovations, the tendency of population aging in China has proposed challenges in domestic healthcare system. Due to widespread ignorance about this disease and lack of early prophylactic measures, the morbidity of osteoporosis appears to be an increasing trend especially among elderly women in the past decade [1, 2]. As the result of severe osteoporosis, old patient often came to hospital with the complaint of debilitating back pain and spinal deformity after a slight trauma or no trauma, and then a moderate or severe vertebral compression fracture would be found by radiological examination. Diagnosed as osteoporotic vertebral compression fractures (OVCF), patients might be suggested to receive conservative treatment, which means continuous resting on bed in most cases. Nonetheless, this treatment might lead to related complications, such as deep vein thrombosis in lower extremities, muscle atrophy, decubitus ulcers, pulmonary or urinary infections [3,6]. On the other hand, pedicle screws fixation, as another common treatment for vertebral fracture, could also be performed to treat OVCF with high risk of surgical complication due to osteoporosis (e.g., screw misplacement, screw pullout, screw loosening and so on) [7, 8]. In addition, poor cardiac and pulmonary conditions are common healthy issues in this population after a surgery under general anesthesia. Under the circumstances, percutaneous kyphoplasty (PKP) was developed as an optimal solution for OVCF patients.

PKP can reinstate vertebral body height, alleviate kyphosis, restore the spinal stability, provide a rapid pain relief and involve simple manipulation techniques. It is generally believed as a minimally invasive and safe intervention, especially for old patients with poor health, because all its manipulation could be finished quickly and accurately under local anesthesia [9,11]. Despite these advantages, PKP also had some drawbacks, such as unsatisfactory reduction, cement leakage, postoperative height loss, adjacent vertebral fracture and so on [12,14]. In recent years, three different approaches raise peoples attention in this field: unilateral transpedicular (UTP), bilateral transpedicular (BTP) and unilateral transverse process-pedicular approaches (UTPP). There is a controversy in the effectiveness and complication among these three approaches [15,18]. Compared to BTP PKP, UTP PKP is considered to have similar surgical outcome but shorter surgery time and lower cost. However, it is necessary to note that unilateral cement injection might lead to spinal stress asymmetry, resulting in high risk of adjacent segments fracture and secondary scoliosis. UTTP PKP, a modified approach of UTP PKP, can deal with the issues mentioned above: the puncture pathway is started at vertebral lamina laterally to pedicle with an extreme extraversion angle. In this approach, the tip of injection needle can reach the midline area on coronal section, which makes the cement symmetrically distributed. Although several studies have focused on the efficacy between two approaches, there is limited research on comparison of those three techniques of PKP. In order to fill in the gap of this field, a retrospective study was performed to compare the surgical outcome and advantage among UTP PKP, BTP PKP and UTTP PKP.

## Methods

### Subjects

The clinical data of participants with thoracolumbar compression fractures were collected in [official title of the hospital] from January 2017 to December 2019. Inclusion criteria includes (1) age of participants should be greater than 60 (2) Patient used to be diagnosed as thoracic or lumbar vertebral fracture secondary to osteoporosis and DXA examination t<2.5; (3) Fracture was confirmed to be fresh by MRI (or was confirmed by ECT if stents was implanted in past); (4) Height loss of the fracture vertebrae was more than 15%, and VAS score was more than 5 (5) PKP was received under local anesthesia, and entire follow-up data were collected. Exclusion criteria was defined as: (1) Vertebral fracture was identified to be secondary to vertebral tumor or infection; (2) Posterior wall of the fractured vertebra was checked by computed tomography (CT) scan to be incomplete, resulting in high risk of cement leakage into spinal canal. (3) patients with history of severe neurological disorders, such as Alzheimers disease, vascular dementia and so on.

### Surgical procedures

**Fig. 1:**
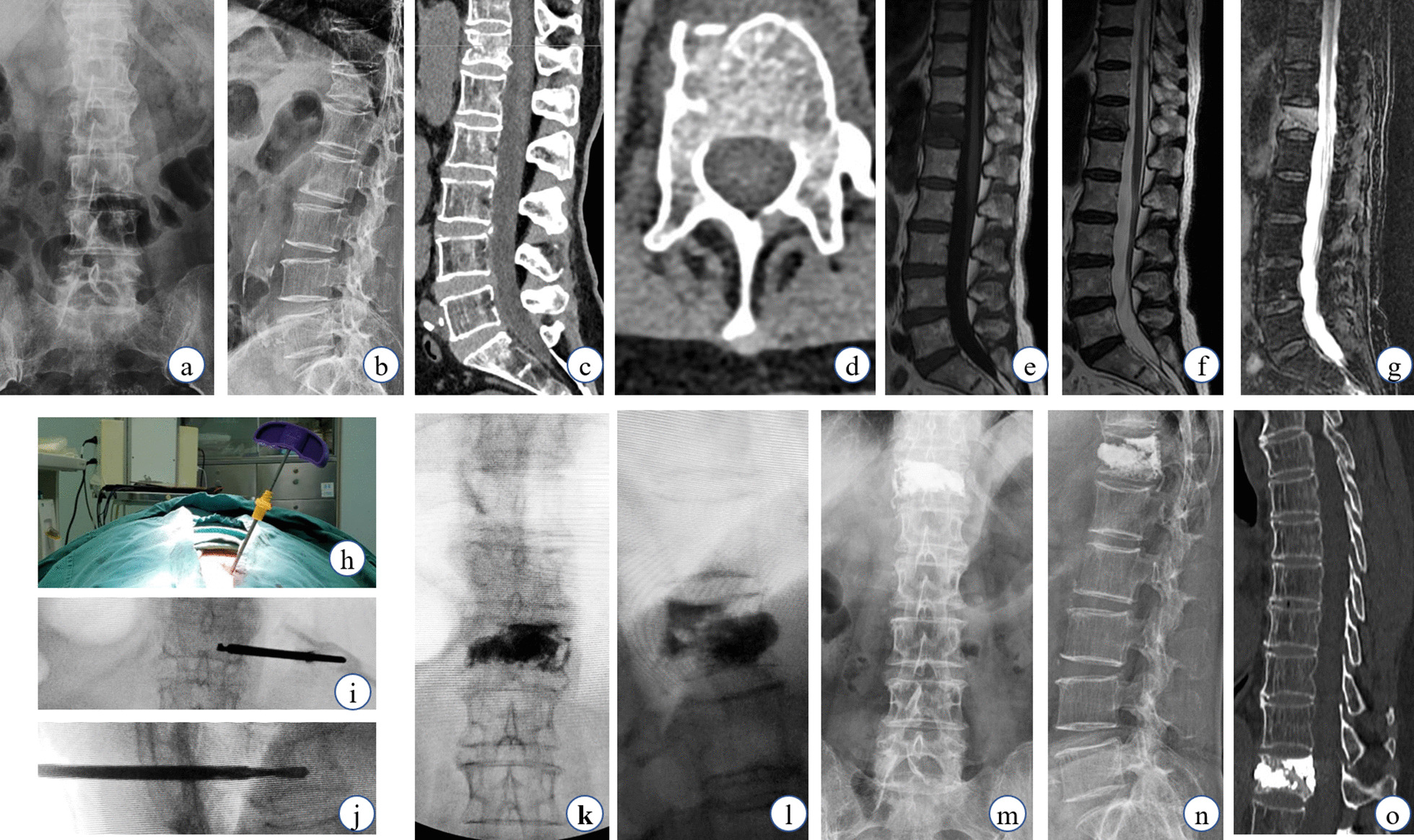
The clinical data of a 72-year-old female OVCF patient with UTTP PKP surgery. The fresh fracture was located at T12 by X-Ray (**a**, **b**), CT scan (**c**, **d**) and MRI (**eg**). And it could be also observed from CT scan that the anterior wall was broken in this vertebra. Forty-degree extraversion angle was used (**h**) and the tip of trocar reached the midline area (**i**, **j**). T12 vertebra was almost filled up with cement on the fluoroscopy and a little cement leaked from the anterior wall of vertebral body (**k**, **l**). Symptom relief and correction of spinal deformity were taken effect immediately after surgery. At the follow-up one month later, symptom was further relieve and no re-collapse was observed (**mo**)

**Fig. 2:**
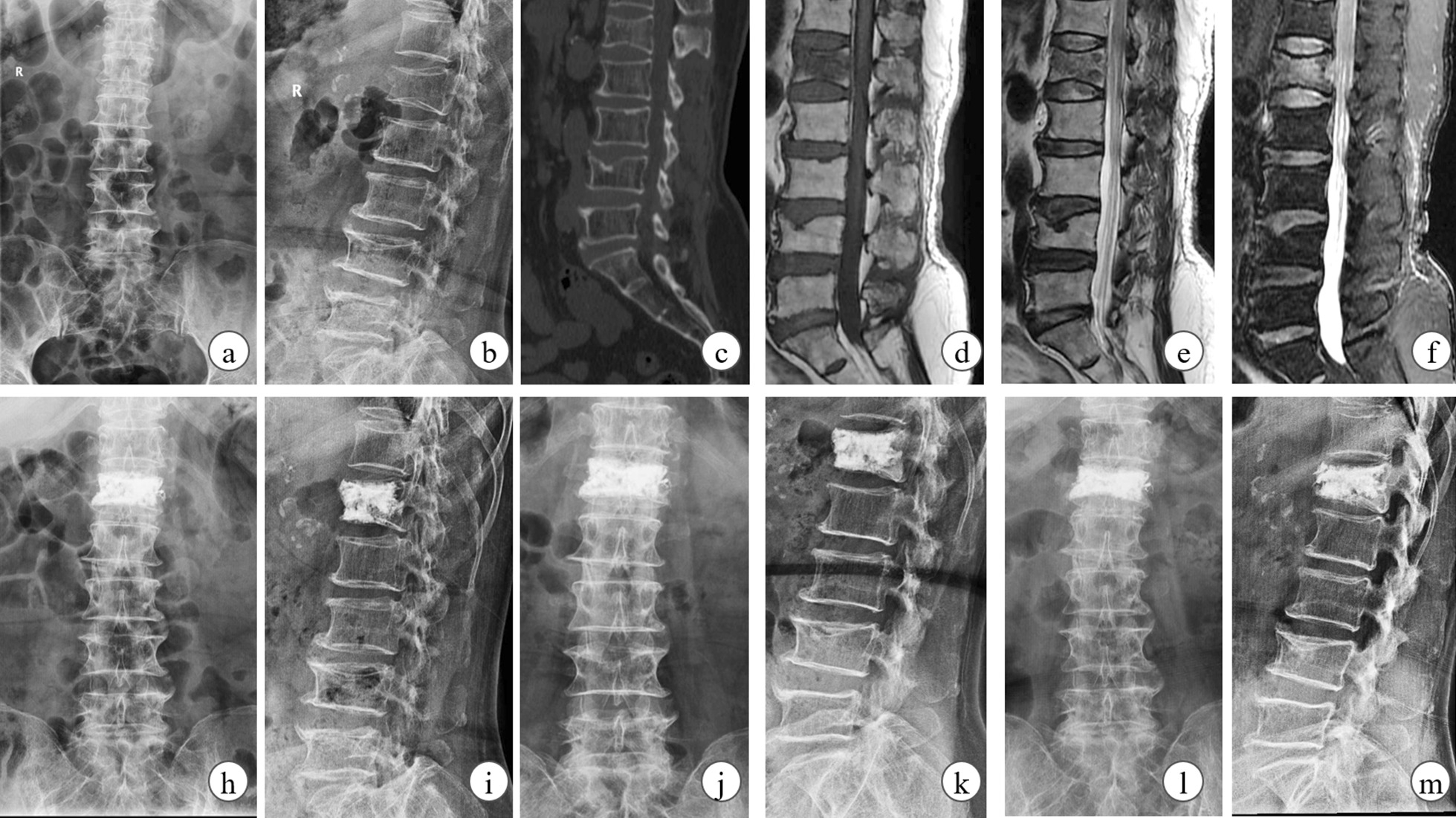
The clinical data of a 70-year-old female OVCF patient with BTP PKP surgery. The fresh fracture was located at L1 by X-Ray (**a**, **b**), CT scan (**c**) and MRI (**df**). L1 vertebra was filled up at two sides with cement and symptom was almost relieve on-month-postoperatively (**h**, **i**). Then slight collapse was observed at follow-ups on X-ray, although the cement volume and distribution were satisfied (**jm**)

**Fig. 3:**
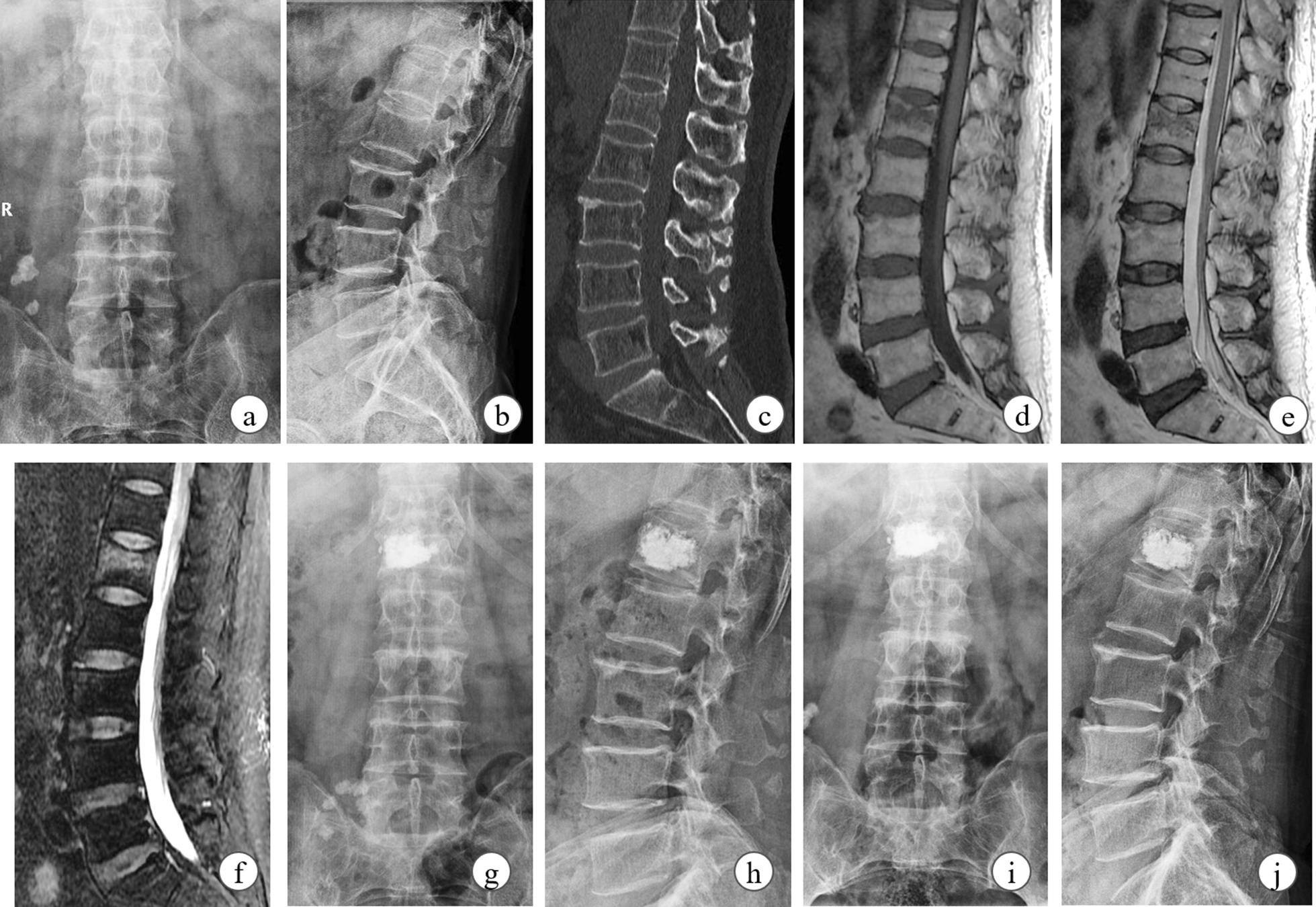
The clinical data of a 68-year-old male OVCF patient with UTP PKP surgery. The patient with the main complaint of severe back pain was diagnosed by radiographical examinations (**af**) as OVCF at L1 segment. At the first follow-up, cement was unilaterally distributed but vertebral height was almost restored (**g**, **h**). No further obvious collapse and symptom recurrence was noted at 1-year postoperative follow-up (**i**, **j**)

**Fig. 4:**
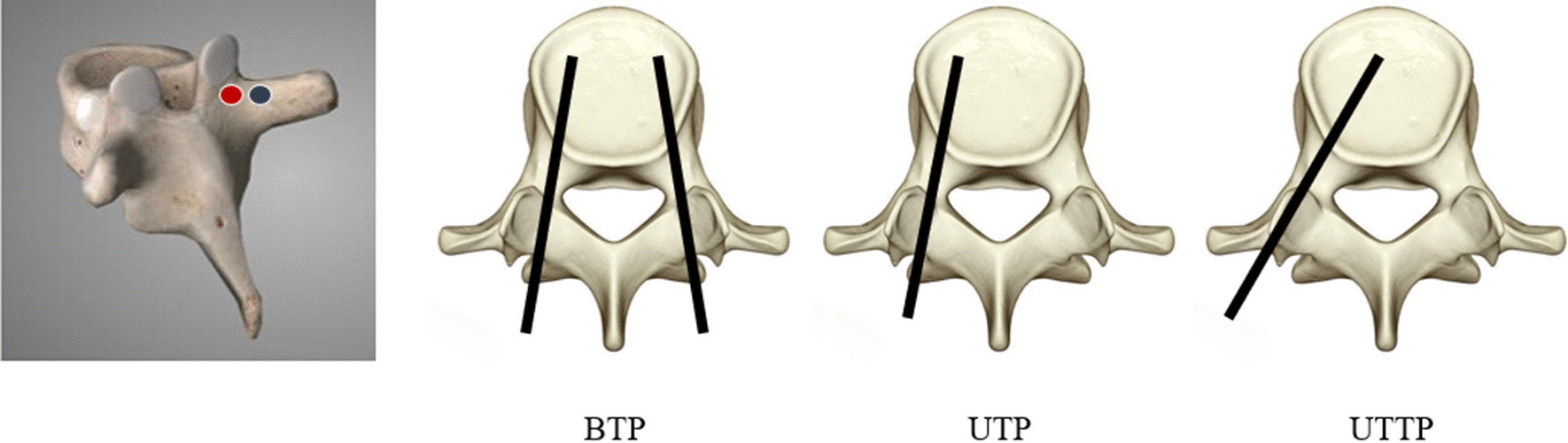
The puncture point of UTP and UTTP. The red point was UTP and blue point was UTTP. Then the extraversion angle of three different approach was performed as preview figure

Each patient in this study received PKP surgery within 2days after the fracture was identified to be fresh. All procedures were performed by Senior spinal surgeons under local anesthesia. Patients were placed in prone position during surgery. A set of bolsters were used to keep abdomen suspended, and then the fractured vertebrae was located and marked by a C-arm fluoroscopy (GE company from USA, oec 9800series). Before the surgery, a gentle force was given at the marked area to make compressed vertebrae overextend and basically reduced. Trocar and cannula systems (KMC; KINETIC MEDICAL Co. LTD, Shanghai, China) were used in three PKP groups. In transpedicular approach, the trocar was punctured at the lateral margin of pedicle with a 20 or so extraversion angle, while the sagittal direction paralleled to the upper endplate. Next, the trocar was substituted by cannula under the help of guide pin. The kyphoplasty balloon was inserted through the cannula and pushed forward to the anterior part of the vertebral body. Contrast agent was pushed meticulously into balloon until the vertebral height was almost restored or the pressure was too high. After all the procedures above, the compressed vertebrae were reduced, and then polymethyl-methacrylate was injected carefully into the vertebrae after the withdrawal of kyphoplasty balloon (Fig. 1). The injection would be terminated if the cement area was no longer enlarged, or cement leakage was observed (Figs. 2, 3). In transverse process-pedicular approach, the puncture point was settled at 5mm laterally when compared with transpedicular approach and the extraversion angle needed to rise up to about 40. Under the ideal condition, the tip of trocar could reach the medial margin of pedicle on the coronal fluoroscopy when it progressed to the posterior wall of vertebral body on the sagittal fluoroscopy. Subsequently, balloon dilation and cement injection could be performed at midline and anterior three fourths of vertebrae (Fig. 1). The remaining procedure of transverse process-pedicle approach was similar as transpedicular approach. All the surgical procedures were performed as Fig.4 under the monitoring of C-arm fluoroscopy. All patients in three groups were prescribed to rest in bed within the following 24h. After surgery, anti- osteoporosis treatment was given for each patient to avoid adjacent vertebral fracture.

### Symptoms and radiological evaluation

Symptom and radiographical evaluation were performed preoperatively, one-month postoperatively, 1-year postoperatively and follow-ups. And follow-ups were repeated every year. Visual Analogue Scale Score (VAS) and Oswestry Disability Index (ODI) scores were used to quantify the extent of clinical symptom. Anterior vertebral height was used to determine the severity of vertebral compression. The vertebral deformity was measured by Cobb method from the upper endplate to the lower endplate of the fractured vertebra on the coronal and sagittal X-ray, respectively. The improvement rate in VAS, ODI, vertebral height and sagittal Cobb angle was defined as the postoperative improvement value divided by the preoperative one. As for sagittal Cobb angel of fractured vertebra, positive value was used to describe kyphosis, whereas negative value indicated lordosis.

### Statistical analysis

The basic health status and surgical indexes were recorded for each patient. All data were analyzed with IBM SPSS 26.0, IBM Corp., Armonk, NY. Pair sample t test was performed to evaluate the different impacts of PKP approaches on the pain relief and deformity correction. After variance was proved to be homogeneous by Levenes test, one-way ANOVA was used to compare variances among three different approaches. And Chi-square test was used to compare frequency. The alpha-level was set at 0.05.

## Results

Totally 447 patients were included with an average age of 76.67.2years old. Of them, 133 were male and the remaining were female. Four hundred and ninety-nine vertebrae were confirmed to be freshly fractured in these patients, including double fractures in 46 and triple in three. Of these fractured vertebrae, 176 were located at thoracic spine and 323 were lumbar. Of those patients, 15 were accompanied with limb fracture and two with pelvic fracture. There were 252 patients accompanied with hypertension, 232 patients with chronic cerebral infarction and 142 patients with chronic heart disease. Patients were divided into three groups according to PKP approaches: UTPP group, UTP group and BTP group.

Our results were showed in detail in Table 1. The gender and average age in UTTP, UTP. BTP groups were (not) statistically significant (p=0.041, p=0.432, respectively). Also, there was no significant difference of the bone mineral density among those three groups (p=0.109). Compared with the other two groups, UTP showed significantly shorter surgical duration (p<0.001), lower cement volume (p<0.001) but higher cement leakage proportion (p=0.044). Additionally, we did not find significant difference of those indexes between UTPP and BTP group.

**Table 1:** The general data of patients in this study

	Improved unilateral group	Unilateral group	Bilateral group
No. of patients	135	211	101
No. of vertebral bodies (units)	148	229	122
Sex(male/female)	28/107	65/146	25/76
Age	76.87.5	76.76.9	75.87.5
Bone density (t value)	3.050.57	3.150.64	3.000.78
Surgery duration/min	33.510.3	22.48.0**	35.312.1
Intraoperative bone cement amount/ml	5.61.3	4.21.2**	5.51.0
VAS			
Preoperative	7.531.18	7.131.11	7.621.07
Postoperative day 1	3.320.84 (55.112.2)	3.210.64 (54.210.5)	3.160.84 (55.512.3)
Last follow-up	2.320.77 (68.312.0)	2.440.85 (65.111.2)	2.330.54 (68.69.8)
ODI score			
Preoperative	34.76.0	33.86.5	35.15.8
Postoperative day 1	13.64.5 (60.113.4)	18.64.1 (59.311.7)	17.73.8 (60.513.9)
Last follow-up	9.64.1 (71.213.7)	17.13.8 (72.414.6)	16.83.6 (74.110.9)
Cobbs Angle (Coronal)			
Preoperative	2.22.3	6.53.2	2.42.3
Postoperative day 1	1.72.6	1.92.5	2.12.1
Last follow-up	1.92.5	2.02.6	2.02.0
Cobbs Angle (Sagittal)			
Preoperative	17.62.1	17.42.7	17.12.2
Postoperative day 1	9.62.3 (46.27.3)	9.83.4 (45.014.1)	9.22.5 (46.78.3)
Last follow-up	10.42.5 (41.28.4)	10.53.3 (40.413.8)	10.12.8 (41.511.8)
Vertebral height/mm			
Preoperative	17.31.8	17.62.9	17.52.2
Postoperative	19.31.9 (11.75.0)	19.22.2 (11.85.0)	19.52.3 (11.64.9)
Last follow-up	18.62.0 (7.36.9)	18.42.4 (7.06.5)	19.22.1 (9.73.2)
Bone cement Leakage/ per vertebra	24 (16.2%)	65 (28.4%)**	19 (15.6%)

The results of pair sample t test showed that immediate significant improvement was observed in terms of both VAS score, ODI score, anterior vertebral height and sagittal Cobb angle within 1week after PKP in three groups, but a slight curative effect loss was noted in terms of vertebral height and sagittal Cobb angle at the last follow-up. Moreover, no significant statistical difference was found in terms of improvement rate of VAS scores, ODI scores, vertebral height and sagittal Cobb angle among three groups, although it seemed that UTP PKP had a worse effect on symptom relief than the two other approaches. Besides, it was notable that the a significantly higher coronal Cobb angle was observed in UTP group, and a about 4 coronal correction was found after UTP PKP.

## Discussion

**Fig. 5:**
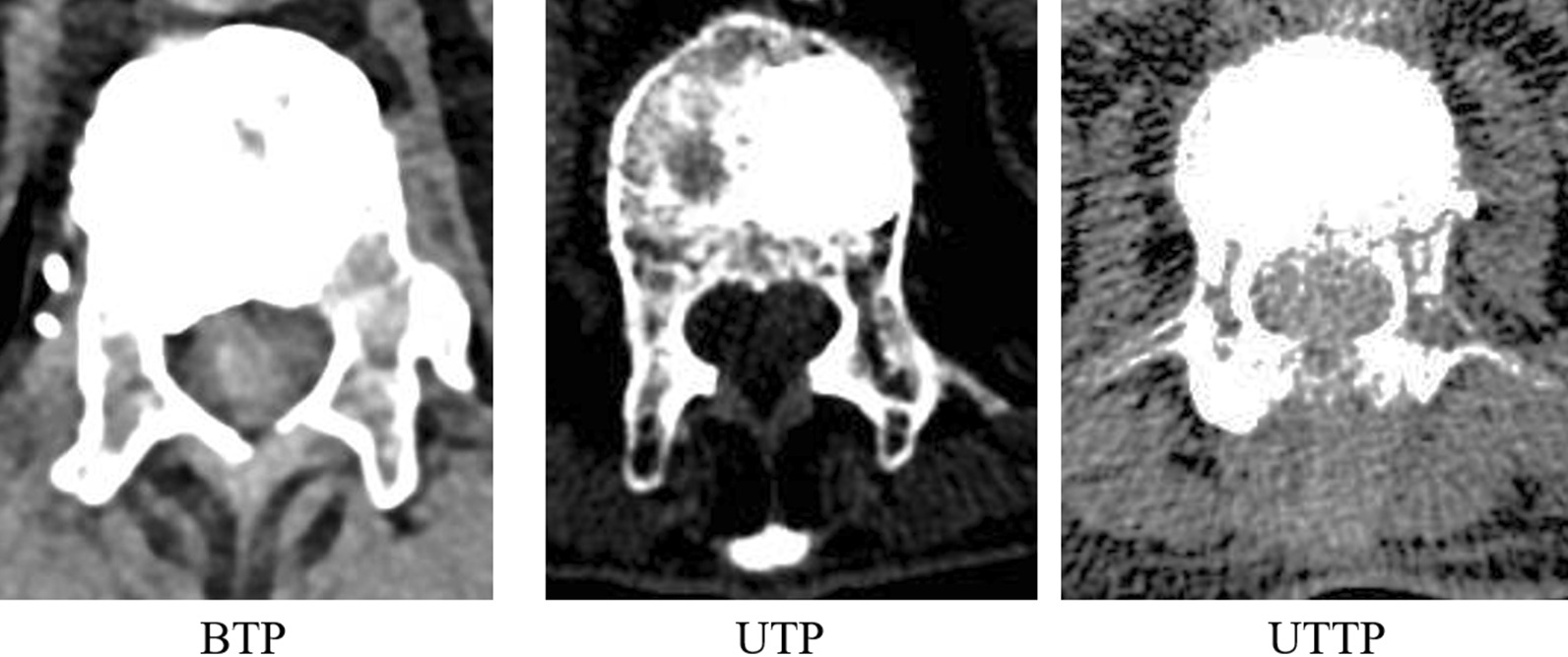
The coronal section of fractured vertebrae in patients with three different surgical approach. It could be noted that cement was eccentrically distributed in patients with UTP and then cement distributed bilaterally in patients with both BTP and UTTP

Percutaneous kyphoplasty (PKP), mainly including transpedicular approach and transverse process-pedicle, was a common minimally invasive surgical technique for the treatment of OVCF due to its outstanding therapeutic effect and simple manipulation. Transpedicular approaches of PKP were frequently discussed and compared in the preview related literatures [15,18]. A multi-center retrospective study conducted by Yilmaz et al. [19] found that significantly shorter surgery time, less bone cement amount, lower complication incidence but similar radiographical correction and symptom relief were noticed with unilateral transpedicular PKP when compared with bilateral transpedicular approach. Another study conducted by Tang et al. [20] also showed that unilateral trans-pedicular PKP had similar surgical efficiency but shorter operative time, lower hospital cost and less radiation exposure. Although the consensus has been reached by preview studies that unilateral pedicular approach PKP had many advantages, only limited amount of researchers recommended it as a preferred treatment for OVCF. It might be contributed to the following defects of unilateral transpedicular approach [21, 22]: First of all, asymmetrical vertebral restoration caused by unilateral balloon dilatation and asymmetrical distribution of bone cement would elevate the risk of contralateral vertebral collapse, resulting in the formation of wedge-shaped vertebral body and finally iatrogenic scoliosis. Next, bone cement leakage was prone to take place due to the limited volume of unilateral distribution. Furthermore, the balloon could not get to the anterior-midline part of vertebrae due to small extraversion angle, which might lead to the unsatisfactory reduction of the anterior vertebral body. To remedy these defects, some modifications were made on the unilateral trans-pedicle approach: First, the puncture point was translocated at least 5mm laterally from that of transpedicular approach. Second, the extraversion angle should be modified to about 40 so that the trocar could cross the outer wall of the pedicle, and eventually graze the inner wall when going into vertebral body. Through this approach, the balloon could dilatate in midline area and reduce the compressed vertebra symmetrically (just as Fig.5). Meanwhile, bone cement could be injected at this area and prone to be distributed bilaterally. Yan et al. [23] compared the outcomes between transverse process-pedicular PKP and bilateral pedicular PKP. Those researchers found that transverse process-pedicular PKP provided a similar symptom relief as control group but had significantly lower surgery duration and less radiation exposure dose. Another study by the same research team [24] showed that patients in the transverse process-pedicular PKP group had a better kyphosis correction due to the location of the balloon and bone cement injection than bilateral transpedicular PKP. Wang et al. [25] pointed out that the unilateral transverse process-pedicular PKP group had a similar clinical outcome as the conventional group, but a wider and symmetrical cement distribution, which contributes to in a better stress distribution. In previous literatures, although a few comparative studies between two approached of unilateral PKP were performed, comparison study among the three different surgical approaches of PKP was rarely reported. Therefore, in order to have a better understanding in this field, this study reviewed the clinical date of cases receiving PKP to determine the advantages, disadvantages and characteristics of the three different surgical approaches.

The results of pair sample and independent sample t test in our study indicated that, under similar preoperative general conditions, all three approaches of PKP could relieve pain, correct kyphosis and restore their self-care ability immediately, and no significant difference was found amongst the three surgical techniques in terms of symptom and kyphosis improvement rate. This finding illustrated that cement injection could restore the stability of fractured vertebrae in each of three approaches. Compared with empirical studies, we also found shorter surgical time in unilateral transpedicular PKP group. Although no significant difference of surgical time was found between the remaining two groups, the unilateral transverse process-pedicular PKP has a shorter operative time than bilateral transpedicular group. This phenomenon might be attributed to the low proficiency of the new surgical technique, which requires larger sample size and further studies to prove. When unilateral transverse process-pedicular PKP was performed, fluoroscopy was more frequently taken to confirm the location of trocar, but the surgical time should be drastically shortened with its proficiency developing. Meanwhile, a significantly higher cement leakage and lower cement volume were found in unilateral transpedicular PKP groups. In this group, the cement was injected from one side of vertebra and difficult to flow into the other side, leading to high proportion of only half cement distribution on fluoroscopy. In order to avoid this issue, cement should be injected quickly and more, which was also the main cause of cement leakage and shortened the surgical time. Besides, we found that part of patients in our study had an asymmetrical compression of vertebral height on the coronal section, and significantly higher coronal Cobb angle was found in unilateral transpedicular PKP group than two other groups, indicating this kind of PKP was prone to be chosen as the prior treatment for patients with asymmetrical vertebral compression in order to correct the spinal deformity at the same time. The results of our study showed that a significant improvement (4 or so) of coronal spinal deformity was noted after unilateral transpedicular PKP, implying this approach of PKP had a slight corrective ability of spinal deformity. In our opinion, it was very important for patients with degenerative scoliosis to avoid the acute progression of spinal deformity due to vertebral compression fracture.This finding was frequently ignored in preview studies.

Although positive results were found in our study, some shortcomings were also listed as follow. Firstly, small sample size, selection bias and limited follow-up duration might lead to the deviation of results. Secondly, these three surgical approaches were started from different time in our center, and surgical approaches were suggested according to their health and economical status, but final decision was made by themselves, which might interfere the result of this study. Last but not least, although both surgeon and measurers were skilled orthopedic surgeons, there were certain individual differences, which may lead to the deviation of the study results. Further work will be done to fill up the defect in future.

## Conclusion

Our research showed that unilateral transverse process-pedicular PKP could achieve similar symptoms relief and kyphosis correction as unilateral and bilateral transpedicular PKP. However, it had shorter surgical time and less radio exposure than bilateral transpedicular PKP, lower risk of cement leakage and higher proportion of bilaterally cement distribution than unilateral transpedicular PKP. It seemed to be a better choice for patients with OVCF. In addition, we found that unilateral transpedicular PKP was especially fit for OVCF patients with asymmetrical vertebral compression due to its slight corrective ability for spinal deformity.

## Data Availability

The datasets generated or analysed during the current study are not publicly available due there were other research plan in future but are available from the corresponding author on reasonable request.
